# Involvement of Polyamine Oxidase-Produced Hydrogen Peroxide during Coleorhiza-Limited Germination of Rice Seeds

**DOI:** 10.3389/fpls.2016.01219

**Published:** 2016-08-12

**Authors:** Bing-Xian Chen, Wen-Yan Li, Yin-Tao Gao, Zhong-Jian Chen, Wei-Na Zhang, Qin-Jian Liu, Zhuang Chen

**Affiliations:** Argo-biological Gene Research Center, Guangdong Academy of Agricultural Sciences Guangzhou, China

**Keywords:** seed germination, polyamine oxidases, hydrogen peroxide, *Oryza sativa*, *OsPAO5*, gene expression, *in silico* analysis

## Abstract

Seed germination is a complicated biological process that requires regulated enzymatic and non-enzymatic reactions. The action of polyamine oxidase (PAO) produces hydrogen peroxide (H_2_O_2_), which promotes dicot seed germination. However, whether and, if so, how PAOs regulate monocot seed germination via H_2_O_2_ production is unclear. Herein, we report that the coleorhiza is the main physical barrier to radicle protrusion during germination of rice seed (a monocot seed) and that it does so in a manner similar to that of dicot seed micropylar endosperm. We found that H_2_O_2_ specifically and steadily accumulated in the coleorhizae and radicles of germinating rice seeds and was accompanied by increased PAO activity as the germination percentage increased. These physiological indexes were strongly decreased in number by guazatine, a PAO inhibitor. We also identified 11 PAO homologs (*OsPAO1–11*) in the rice genome, which could be classified into four subfamilies (I, IIa, IIb, and III). The OsPAO genes in subfamilies I, IIa, and IIb (*OsPAO1–7*) encode PAOs, whereas those in subfamily III (*OsPAO8–11*) encode histone lysine-specific demethylases. *In silico*-characterized expression profiles of *OsPAO1–7* and those determined by qPCR revealed that *OsPAO5* is markedly upregulated in imbibed seeds compared with dry seeds and that its transcript accumulated to a higher level in embryos than in the endosperm. Moreover, its transcriptional abundance increased gradually during seed germination in water and was inhibited by 5 mM guazatine. Taken together, these results suggest that PAO-generated H_2_O_2_ is involved in coleorhiza-limited rice seed germination and that *OsPAO5* expression accounts for most PAO expression and activity during rice seed germination. These findings should facilitate further study of PAOs and provide valuable information for functional validation of these proteins during seed germination of monocot cereals.

## Introduction

Seed germination involves complex physiological and biochemical processes, e.g., signal transduction and gene expression regulation (Bewley, 1997; Finch-Savage and Leubner-Metzger, 2006; Gomes and Garcia, 2013; He and Yang, 2013; Han and Yang, 2015). For dicot seeds, e.g., Arabidopsis (*Arabidopsis thaliana*), tomato (*Solanum lycopersicum*), cress (*Lepidium sativum*) and tobacco (*Nicotiana tabacum*), their micropylar endosperm (usually denoted the endosperm cap) is mechanically strong and acts as a physical barrier to the completion of germination (Leubner-Metzger and Meins, 2000; Nonogaki et al., 2000; Müller et al., 2006; Iglesias-Fernandez and Matilla, 2010; Nonogaki, 2014). In monocot seeds, particularly those of cereals, e.g., barley (*Hordeum vulgare*), rice (*Oryza sativa*), and purple false brome (*Brachypodium distachyon*), the coleorhiza, which is a non-vascularized multicellular embryonic tissue covering the seminal seed root, is believed to regulate emergence of the radicle during germination (Millar et al., 2006; Gonzalez-Calle et al., 2015). It has been assumed that dicot seed germination is controlled by the mechanical force of the imbibed, elongating radicle on the endosperm cap and by inherent cap weakening driven by enzymatic (i.e., endo-β-1,4-mannanases and pectin methylesterases) and non-enzymatic reactions [e.g., those involving reactive oxygen species (ROS); Nonogaki et al., 2010; Zhang et al., 2014; Scheler et al., 2015; Chen et al., 2016]. Given the physical and chemical similarities of dicot and monocot seed structures, logically similar enzymatic and non-enzymatic mechanisms would be required during monocot seed germination.

Previous studies have shown that ROS, e.g., the superoxide radical (O_2_^-^), hydrogen peroxide (H_2_O_2_), and the hydroxyl radical (^⋅^OH), function as positive and negative signaling molecules during seed germination (D’Autreaux and Toledano, 2007; Tripathy and OelMüller, 2012; Gomes and Garcia, 2013). The involvement of ROS in seeds, e.g., endosperm weakening, mobilization of seed reserves, protection against pathogens, and programmed cell death, is well-known (El-Maarouf-Bouteau and Bailly, 2008; Gomes and Garcia, 2013). H_2_O_2_ serves as a molecular ‘hub’ during ROS-mediated signaling in plants; specifically, in seeds it is important for cell wall loosening, which is necessary for seed germination (Quan et al., 2008; Gomes and Garcia, 2013; Wojtyla et al., 2016). The ROS with the greatest reactivity and shortest life span is ^⋅^OH, which is formed from O_2_^-^ and H_2_O_2_ in the apoplast by the action of cell wall peroxidases and can directly degrade cell wall polysaccharides, thereby loosening the cell wall (Schweikert et al., 2000; Müller et al., 2009).

Polyamine (PA) catabolism is an important pathway for H_2_O_2_ generation. Polyamine oxidases (PAOs) generate H_2_O_2_ by oxidative degradation of the PAs putrescine (Put), spermidine (Spd), and spermine (Spm). PAs are aliphatic amines of relatively small molecular mass involved in various physiological processes in plants, e.g., growth, development, and stress responses (Alcázar et al., 2010; Mattoo et al., 2010). The PAOs, copper-dependent diamine oxidases (EC 1.4.3.6), and flavin adenine dinucleotide-associated PAOs (EC 1.5.3.11) catalyze oxidation of deaminated moieties at primary and secondary amino groups while generating H_2_O_2_ as a product (Kusano et al., 2008; Moschou et al., 2012; Planas-Portell et al., 2013). Based on the chemical structures of their reaction products, PAOs are classified as: (i) those responsible for terminal catabolism of PAs, during which the carbon on the endo-side of the N_4_-nitrogens of Spd and Spm is oxidized-these PAOs are only found in plants (Cervelli et al., 2006; Moschou et al., 2008b; Liu et al., 2014a); (ii) those responsible for back-conversion of PAs by oxidizing the carbon on the exo-side of the secondary amino of N_1_-acetylderivatives in animals and non-acetylated PAs in plants (Tavladoraki et al., 2006; Kamada-Nobusada et al., 2008; Moschou et al., 2008b; Ono et al., 2012); (iii) those that contain a PAO domain but do not deaminate PAs; instead they demethylate histone H3K4 in animals and plants (Shi et al., 2004; Spedaletti et al., 2008; Mosammaparast and Shi, 2010; Luo et al., 2014; Prakash et al., 2014).

The biological significance and physiological functions of PAOs from several organisms have been characterized. For example, the PAOs in the monocots maize (*Zea mays*; *ZmPAO1*), barley (*HvPAO1* and *HvPAO2*), and rice (*OsPAO7*) are involved in the terminal catabolism of PAs, and they oxidize the carbon at the endo-side of the N_4_ of Spm and Spd to produce *N*-(3-aminopropyl)-4-aminobutanal and 4-aminobutanal, respectively, 1,3-diaminopropane, and H_2_O_2_ (Tavladoraki et al., 1998; Cona et al., 2005; Cervelli et al., 2006; Liu et al., 2014a). The five PAOs in *Arabidopsis* (*AtPAO1–5*) and four of seven PAOs in rice (*OsPAO1*, *OsPAO3*, *OsPAO4*, and *OsPAO5*) catalyze the back conversion of Spm (or T-Spm) to Spd and/or Put in a manner similar to that of animal PAOs/SMOs (Kamada-Nobusada et al., 2008; Moschou et al., 2008c; Takahashi et al., 2010; Fincato et al., 2011; Liu et al., 2014b). Moreover, the aforementioned PAOs are found in different subcellular locations, during different developmental stages, or have different tissue-specific expression profiles. For example, *ZmPAO1*, *HvPAO1/2*, and *OsPAO7*, involved in terminal catabolism of PAs, are located at the edge of the plant cell although *HvPAO1/2* and *OsPAO7* expression is greatest in ear organs, sterile spikelets, and anthers (Tavladoraki et al., 1998; Cona et al., 2005; Cervelli et al., 2006; Ono et al., 2012; Liu et al., 2014a). In contrast, *AtPAO1–5* and *OsPAO1/3–5*, involved in back-conversion of Spm and T-Spm, are present in the cytoplasm and peroxisomes, with the *OsPAO3–5* transcription levels greatest in 2-weeks-old seedlings and the *OsPAO1* expression lowest (Fincato et al., 2011; Ono et al., 2012). In addition, the most abundant transcripts of *AtPAO1/2/3/5* are in flowers, whereas the highest level of *AtPAO4* expression is found in young seedlings, particular in their roots (Takahashi et al., 2010). Furthermore, *AtPAO4* deficiency is induced by alterations in the expression of genes related to drought stress response and flavonoid biosynthesis (Kamada-Nobusada et al., 2008). Interestingly, the third group of PAOs, the *Arabidopsis* and rice homologs of human lysine-specific demethylases, regulate flowering time and, for *Arabidopsis* seed dormancy, by demethylation of histone H3K4 (Shi et al., 2004; Jiang et al., 2007; Spedaletti et al., 2008; Mosammaparast and Shi, 2010; Luo et al., 2014; Shafiq et al., 2014; Zhao et al., 2015).

Although these studies on *Arabidopsis*, maize, barley, and rice PAOs have led to an understanding of their biochemical properties and physiological functions, characterization of PAO functions during rice seed germination has not been undertaken. Recent work has shown that ROS may have a regulatory role during the life stages of seeds, e.g., germination and release from dormancy (Nonogaki, 2014). Therefore, because PAOs generate H_2_O_2_, they may be involved in seed germination via PA catabolic pathways. For the study reported herein, we performed a comprehensive evaluation of the role(s) played by PAOs during germination of rice seeds. Our study included characterizing the morphology of the germinating seeds, a histochemical analysis, quantification of ROS accumulation, measurement of PAO activity, and assessment of PAO gene expression profiles. The results should increase our understanding of the involvement of rice PAOs and their reaction product H_2_O_2_ in coleorhiza-limited seed germination and allow for further studies of the physiological role(s) of the PA catabolic pathways in plants.

## Materials and Methods

### Non-plant Materials

Guazatine, *N*,*N′*-dimethylthiourea (DMTU), nitroblue tetra zolium (NBT), 3, 3-diaminobenzidine hydrochloride (DAB), 3,3*′*,5,5*′*-tetramethylbenzidine (TMB), Spm, Spd, Put, 4-amino antipyrine, *N*,*N′*-dimethylaniline, and horseradish peroxidase were purchased from Sigma-Aldrich. Water used was always doubly distilled.

### Plant Materials and Seed Germination

Rice seeds (*O. sativa* ssp. *japonica* cv. Nipponbare) with the glume removed were placed into a transparent plastic germination box (12 cm × 12 cm × 6 cm) containing two layers of filter paper soaked in water; 5 mM DMTU; 10 mM H_2_O_2_; 5 mM guazatine; 5 mM DMTU plus 10 mM H_2_O_2_; or 5 mM guazatine plus 10 mM H_2_O_2_ (20 mL each). The seeds were incubated in a growth chamber at 28 ± 1°C under a 16-h light/8-h dark photocycle (10,000 lux). Seeds with protruding radicles were regarded as having finished germination and were counted at 6-h intervals from 12 to 48 h. The number of germinated seeds at each time point was converted to a percentage, and the mean value ± SE of three biological replicates of 100 seeds each was calculated. Seeds were photographed using the stereomicroscope (SteREO Lumar V12, Zeiss, Germany).

### Histochemical Localization and Quantification of O_2_^-^ and H_2_O_2_

We used NBT and DAB, respectively, to stain seeds for O_2_^-^ and H_2_O_2_ as described (Zhang et al., 2014; Chen et al., 2016). After rice seeds had imbibed water or 5 mM guazatine for 3, 6, 12, 24, or 48 h, five whole seeds and five half granule seeds containing the embryos were removed and incubated with 1 mM NBT in 10 mM Tris-HCl (pH 7.0), or 1 mg/mL DAB (pH 3.8) at room temperature for 30 min, then washed with double-distilled water, and photographed using the stereomicroscope (SteREO Lumar V12, Zeiss, Germany).

The rate of O_2_^-^ production (nmol O_2_^-^⋅min^-1^⋅g^-1^ FW) and H_2_O_2_ concentration (μmol⋅g^-1^ FW) were spectro photometrically measured as described [fresh weight (FW); Zhang et al., 2014; Chen et al., 2016]. Thirty embryos at each aforementioned imbibition time points were used for each type of measurement, and the mean value ± SE of three biological replicates were calculated.

### Histochemical Detection of Peroxidase Activity

POD activity was detected histochemically by TMB staining as described (Zhang et al., 2014; Chen et al., 2016). Rice seeds were imbibed in water or 5 mM guazatine for the aforementioned five times. Five whole seeds and five half granule seeds containing their embryos were incubated in 0.2% (w/v) TMB, 1 mM H_2_O_2_, 20 mM phosphate (pH 6.5) at room temperature for 30 min, then washed with water, and photographed using the stereomicroscope (SteREO Lumar V12, Zeiss, Germany).

### PAO Activity Assay

Embryos (0.2 g) from whole seeds imbibed for the aforementioned five times were extracted and immediately ground in a TissueLyser-24 (Shanghai Jingxin Industrial Development, Co., Ltd, China) at 4°C in 1.0 mL 0.1 mol/L sodium phosphate (pH 6.5). The homogenates were centrifuged at 10,000 × *g* and 4°C for 20 min. The supernatants were individually transferred into new tubes and centrifuged again at 5,000 × *g* and 4°C for 5 min. The second set of supernatants were assayed for PAO activity. To determine the optimal substrate and wavelength for PAO activity measurements, first the oxidation of Spm, Spd, or Put was observed after horseradish peroxidase oxidation of 4-aminoantipyrine and *N*,*N′*-dimethylaniline monitored between 300 and 800 nm (Su et al., 2006; Tavladoraki et al., 2006; Liu et al., 2014a). The reaction solutions (3.0 mL) each contained 2.5 mL 100 mM sodium phosphate (pH 6.5), 100 mM 4-aminoantipyrine, 1 mM *N*,*N′*-dimethylaniline, 0.1 mL horseradish peroxidase (250 U/mL), 0.2 mL of a crude enzyme extract and 0.2 mL of a substrate (20 mmol/L Spm, Spd, or Put). Assays were initiated by addition of a substrate and incubated at 30°C for 30 min. A_515_ was measured using a Multiskan Spectrum spectrophotometer (Varioskan Flash, Thermo, USA). A 0.01 change in the A_515_ was defined as one enzyme activity unit.

### Identification and Phylogenetic Analysis of a PAO Gene Family

The latest non-redundant set of protein sequences for the monocot, *O. sativa*, and eudicot, *A. thaliana*, were retrieved from the Rice Annotation Project Database^1^ and the *Arabidopsis* Information Resource (TAIR v10.0^2^), respectively. The sequences were incorporated into an in-house database and the procedures described in Li et al. (2014), Chang et al. (2016) were used to identify the rice and *Arabidopsis* PAO homologs, with the one difference that the family specific amino oxidase domain (PF01593) HMM profile was used in the HMM search. Then, after aligning the amino oxidase domain sequences of the identified PAO proteins, they were used to construct a phylogenetic tree as described in Li et al. (2014), Chang et al. (2016).

### *In silico* Expression Profiles (Heat Maps) and Quantitative Real-Time PCR (qPCR) of PAO Homologs

We used the Os_51k microarray data in the Genevestigator V3 database to analyze the expression profile**s** of rice PAO genes, by constructing heat maps from the data sets (Hruz et al., 2008).

To characterize the expression profiles of OsPAO genes by qPCR, 30 embryos from seeds incubated in water or 5 mM guazatine for the aforementioned five imbibition times were extracted and immediately frozen at -80°C. Total RNA was isolated using Column Plant RNAout 2.0 kit reagents (TIANDZ, China) according to the manufacturer’s instructions, and qPCR was performed as described (Li et al., 2014; Chang et al., 2016). The gene-specific primers used (Supplementary Table S3) were designed to avoid conserved regions, introns, and an exon–exon junction. *OsGAPDH1* (RAP-DB ID: Os02g0601300) expression served as the internal control. Mean value ± SE of three biological replicates were calculated.

### Statistical Analysis

Data are presented as the mean ± SE of three replicates. One-way analysis of variance was used to compare mean values, and when significant, differences between individual means were compared with the Fisher’s least-significant difference test. Student’s *t*-test were conducted to evaluated variances in the expression levels of *OsPAO1–7*.

## Results

### Rice Seed Germination is Promoted by Exogenous and PAO-Produced Endogenous H_2_O_2_, But Is Inhibited by DMTU and Guazatine

**FIGURE 1:**
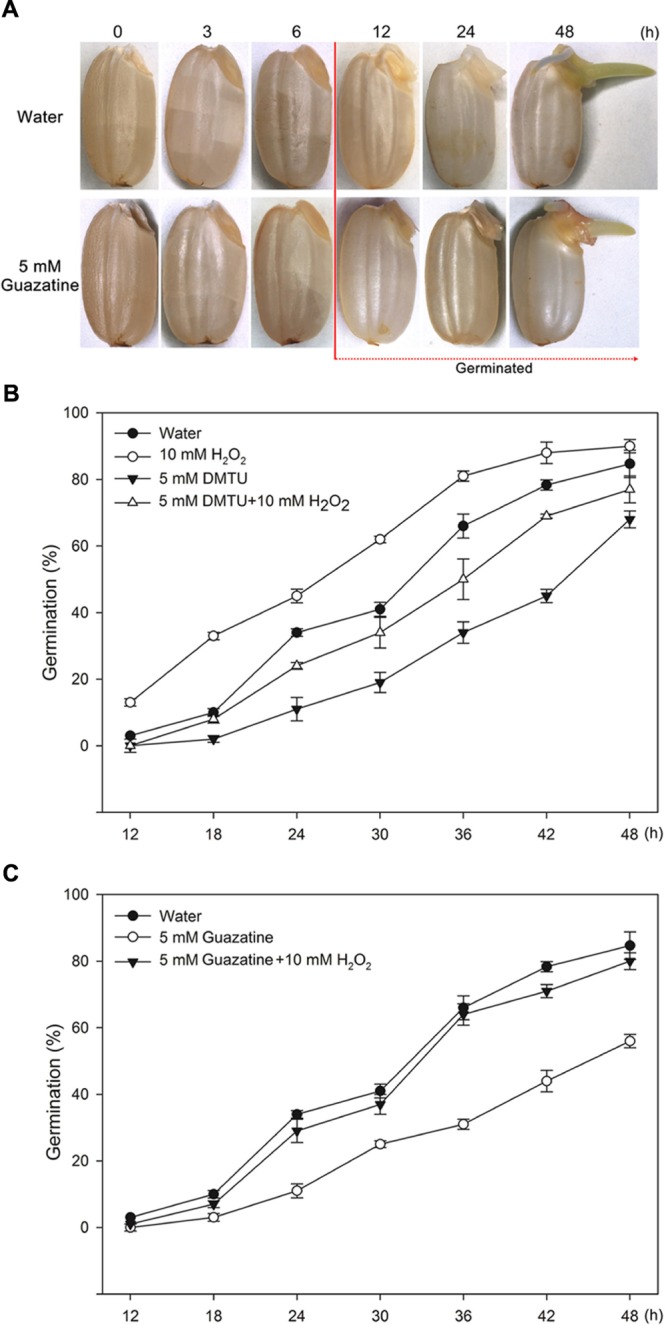
**Morphologies and germination time courses of rice seeds. (A)** Morphologies of rice seeds imbibed in (top) water or in (bottom) 5 mM guazatine. Germination time courses for rice seeds imbibed in **(B)** water, 10 mM H_2_O_2_, 5 mM DMTU, 5 mM DMTU plus 10 mM H_2_O_2_, 5 mM guazatine, or **(C)** water, 5 mM guazatine or 5 mM guazatine plus 10 mM H_2_O_2_. Germination number was scored every 6 h for 48 h, and the results are presented as the cumulative germination percentages. Data are the mean ± SE of three biological replicates of 100 seeds each.

To determine whether PAO production of H_2_O_2_ promotes germination of rice seeds, we characterized the morphology and percentage of germinating seeds that had been imbibed in only water or in aqueous solutions containing exogenously added H_2_O_2_, DMTU (a scavenger for H_2_O_2_), guazatine (a competitive inhibitor of PAOs), H_2_O_2_ and DMTU, or H_2_O_2_ and guazatine at various times (**Figure 1**). The first seeds to complete germination in water did so by 12 h [**Figures 1A(top),B,C]**; 50% of the seeds incubated in water completed germination by 30 h, and 84% within 48 h (**Figures 1B,C**). Germination was promoted by 10 mM H_2_O_2_ but inhibited by 5 mM DMTU (**Figure 1B**). When the seeds were imbibed in 5 mM DMTU plus 10 mM H_2_O_2_, the germination percentage was always less than that for seeds germinated in water alone but greater than that for seed germinated in 5 mM DMTU (**Figure 1B**). Notably, 5 mM guazatine did not introduce a lag period before germination was observable, but reduced the germination percentage and inhibited the growth of the coleoptile and radicle (**Figures 1A,C**). When seeds were imbibed in 5 mM guazatine plus 10 mM H_2_O_2_, the extent of germination was completely recovered at each time point (**Figure 1C**). These results demonstrate that a PAO(s) may promote rice seed germination by producing H_2_O_2_ via oxidative degradation of PAs.

### ROS are Produced and Accumulate in the Embryo and Aleurone Layer of the Rice Seed upon Imbibition in Water, But This Process Is Partially Inhibited by Guazatine

**FIGURE 2:**
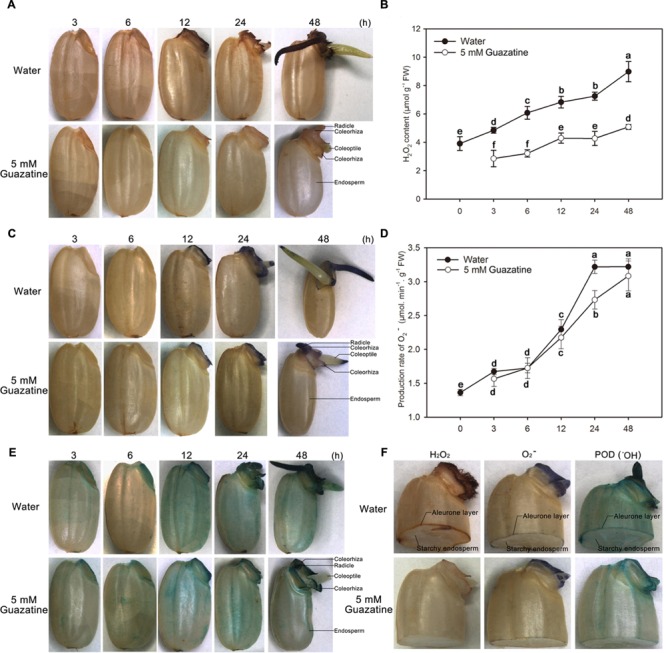
**Histochemical staining and quantification of H_2_O_2_ and O_2_^-^ content, and peroxidase activity during germination of rice seeds in water or guazatine.** Histochemical staining for the location of **(A)** H_2_O_2_ **(C)** O_2_^-^, and **(E)** peroxidase activity in whole rice seeds and in **(F)** embryos of half granule seeds after imbibition of water or 5 mM guazatine. Quantitative determination of **(B)** H_2_O_2_ content and **(D)** O_2_^-^ production in seeds imbibed in water or in 5 mM guazatine. Data are the mean ± SE of three biological replicates of 30 embryos (∼0.1 g total). Means denoted by the same letter did not significantly differ at *P* < 0.05 according to Fisher’s least significant difference test. FW, fresh weight.

To characterize the distribution of ROS in germinating seeds, the presence of H_2_O_2_, O_2_^-^, and POD activity (an indirect measure of the production and accumulation of ^⋅^OH) were detected, respectively, by DAB, NBT, and TMB staining of the aleurone layer and embryo. H_2_O_2_, O_2_^-^, and ^⋅^OH accumulated throughout the time course of the germination period. The embryo and aleurone layer were somewhat stained by all three stains, whereas the starchy endosperm was not (**Figures 2A,C,E,F**).

When seeds imbibed only in water, the whole seed was stained by DAB, with the embryo, especially its radicle, coleorhiza, and coleoptile, most strongly stained (**Figure 2A**). Staining of the embryo increased as the imbibition time increased, except that the coleoptile was only faintly stained at 48 h. Conversely, for seeds imbibed in 5 mM guazatine, their embryos, especially their radicles, were stained to a lesser extent (**Figure 2A**). The H_2_O_2_ content in the embryos was quantified spectrophotometrically (**Figure 2B**), which showed that when seeds were imbibed in water, the H_2_O_2_ content in the embryo increased throughout the imbibition time. However, when seeds were imbibed in 5 mM guazatine, their H_2_O_2_ content increased more slowly and its concentration in the embryos was greatly reduced compared with that for embryos imbibed in water. These results agree with those of the histochemical staining (**Figure 2A**), indicating that guazatine significantly inhibits H_2_O_2_ production in the embryos of germinating rice seeds.

Production and accumulation of O_2_^-^ were also investigated by NBT staining of the embryos of the rice seeds imbibed in water or 5 mM guazatine. When the seeds were imbibed in water, their embryos were stained only after 12 h, and moreover, their coleorhiza, coleoptile, and radicle stained strongly after 12 h. As was found for water imbibition, the embryos were not initially stained when the seeds were imbibed in 5 mM guazatine, but were stained after 12 h (**Figure 2C**). The rate of O_2_^-^ production in the embryos was also quantified spectrophotometrically (**Figure 2D**) and found to increase slowly before 6 h of water imbibition, increase rapidly thereafter, and be maintained between 24 and 48 h. When seeds were imbibed in 5 mM guazatine, however, the rate of O_2_^-^ production was not significantly different to that found for water imbibition throughout most of the experiment. Therefore, unlike H_2_O_2_ production, O_2_^-^ production was not suppressed by guazatine.

Because POD catalysis produces ^⋅^OH (Schopfer et al., 2001; Liszkay et al., 2004; Ren et al., 2008; Gonzalez-Calle et al., 2015), we assessed the POD activity in rice seeds that had been imbibed in water or in 5 mM guazatine by TMB staining (**Figure 2E**). Whole seeds imbibed in water or guazatine were completely TMB stained after 12 h. The intensity of the TMB stain in the embryo increased throughout the imbibition time in water and guazatine. For seeds imbibed in 5 mM guazatine, the intensity of the TMB staining (especially in the embryo) was less than that for those imbibed in water, indicating that guazatine probably reduced POD activity in the rice seeds.

### Activity of Rice PAO(s), for Which Spm Is the Optimal Substrate, Increased Gradually in Embryos of Rice Seeds upon Water Imbibition and Was Intensely Inhibited by Guazatine Imbibition

**FIGURE 3:**
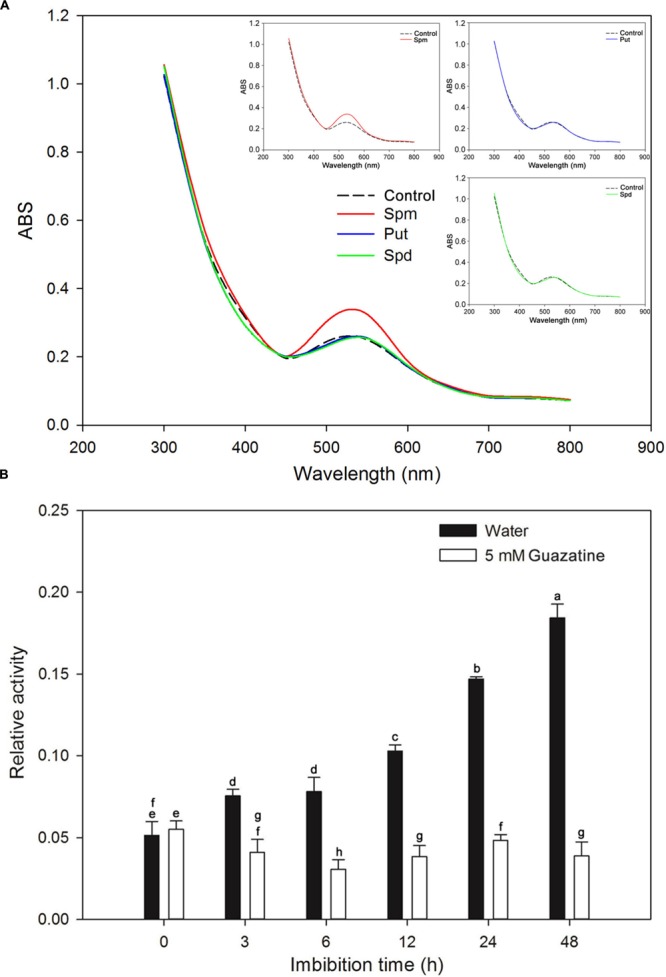
**Identification of the optimal substrate and absorbance peak for crude PAO activity and measurement of PAO activity during rice seed germination. (A)** Substrate specificity and absorbance spectra found for the crude PAO activity in rice seeds after imbibed in water for 12 h. **(B)** Crude PAO activity in rice seeds, imbibed in water or in 5 mM guazatine at 3, 6, 12, 24, and 48 h, was determined using Spm as the substrate. Data are the mean ± SE of three biological replicates of 60 embryos (∼0.2 g total). Means denoted by the same letter did not significantly differ at *P* < 0.05 according to Fisher’s least significant difference test.

A crude PAO embryo extract was assessed for PAO activity. Initially, we determined the substrate specificities and optimum absorption peak for the assay with Spm, Spd, and Put as substrates. When assayed, the crude extract had an absorbance peak centered at 515 nm (**Figure 3A**), a finding similar to that for PAOs from the lateral root of soybean (peak maximum at 555 nm; Su et al., 2006). The maximum activity was obtained for Spm as the substrate (**Figure 3A**). Therefore, Spm was used as the substrate for the time course experiment described below.

We determined the PAO activity in embryos of rice seeds during germination in water and in guazatine (**Figure 3B**). PAO activity in dry seeds (0 time of imbibition) was minimal, but increased in the embryos as the time of imbibition in water increased. Furthermore, by the end of the experiment (48 h) the mean radicle length was ∼1 cm (**Figure 1A**). The data indicate that PAO activity may be important for seed germination and radicle elongation. When the seeds were imbibed in 5 mM guazatine, PAO activity in the embryo decreased strongly, and it was significantly less than in dry seeds, suggesting that guazatine specifically reduced PAO activity in the rice seed during germination.

### Phylogenetic Analysis of PAO Gene Family Indicated 11 PAO Homologs in Rice Were Classified into Four Well-Conserved Subfamilies with Distinct Subcellular Locations, Domain Organizations, and Diversified Functions

**FIGURE 4:**
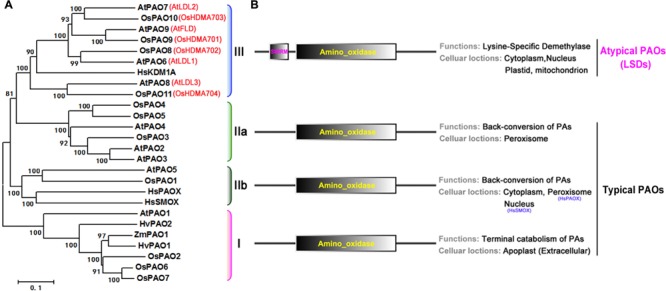
**Phylogenetic tree, predicted locations and functions of PAO protein family. (A)** Unrooted maximum-likelihood phylogenetic tree for rice and *Arabidopsis* PAO protein family constructed based on an amino acid sequence alignment of their amino_oxidase domains. Human, maize, and barley sequences are included. HsPAOX (*Homo sapiens*, ENSP00000278060), HsSMOX (*H. sapiens*, ENSP00000307252), HsKDMA (*H. sapiens*, ENSP00000383042), ZmPAO1 (*Zea mays*, NM_001111636), HvPAO1/2 (*Hordeum vulgare*, AJ298131 and AJ298132). The associated bootstrap values from 1000 replications are given at their nodes, and the branch lengths are drawn to scale. The subfamily members are bracketed by color, and their subfamily numbers, I, IIa, IIb, and III, are shown to the right of the tree. **(B)** Domain organizations (left), and predicted functions and locations (right).

To characterize the phylogenetic relationship among rice and *Arabidopsis* PAO family genes, first a hidden Markov model search was performed to find the sequences related to the family specific amine_oxidase domains (PF01593), and a total of 11 rice and 9 *Arabidopsis* PAO homologs were identified (Supplementary Table S1). Then, an unrooted maximum-likelihood (ML) phylogenetic tree (**Figure 4**) was constructed using these sequences (Supplementary Figure S1). According to the topology and the deep-duplication nodes of the tree, these PAOs can be classified into the four well-known and conserved subfamilies (I, IIa, IIb, and III; **Figure 4A**) with statistical confidence. In addition to the typical amino_oxidase domain found in these proteins, subfamily III also contain a SWIRM (PF04433) domain upstream of the amino oxidase domain (**Figure 4B**). Notably, the subfamily III proteins are not PAOs, but histone lysine-specific demethylases, which catalyze the demethylation of H3K4 histone lysine residues via an FAD-dependent oxidation. These demethylases regulate plant growth and developmental processes, e.g., flowering time and seed dormancy (Spedaletti et al., 2008; Luo et al., 2014; Prakash et al., 2014; Zhao et al., 2015).

Furthermore, we identified the subcellular locations of these proteins with the use of the crop Proteins with Annotated Locations database^3^ and SubCellular Proteomic database^4^, respectively (Supplementary Table S1). The classifications, locations, and functions (Tavladoraki et al., 1998; Cervelli et al., 2006; Jiang et al., 2007; Takahashi et al., 2010; Fincato et al., 2012; Ono et al., 2012; Liu et al., 2014a,b; Luo et al., 2014) of the proteins are summarized in **Figure 4B**. The subfamily I, IIa, and IIb PAOs are FAD-dependent amine oxidases and catalyze the catabolism of PAs. The subfamily I PAOs catalyze the final step in PA catabolism and are located extracellularly in the apoplast, whereas the subfamily IIa and IIb PAOs catalyze the back conversion of PAs and are located in the peroxisome and cytoplasm, respectively. However, the subfamily III proteins, although they have a typical amino oxidase domain, are histone lysine-specific H3K4 demethylases, and are found in many different organelles such as the nucleus, cytoplasm, plastid, and mitochondria. Thus, given their domain organizations and catalytic activities, these proteins can be categorized as typical PAOs (subfamilies I, IIa, and IIb, OsPAO1–7 and AtPAO1–5) and atypical PAOs or lysine-specific demethylases (subfamily III, OsPAO8–11 and AtPAO6–9).

### The Expression Profiles of *OsPAO1–7* Differ Significantly during Germinations, and the Transcript Level of *OsPAO5* Parallels that of PAO Activity and Change in H_2_O_2_ Content in the Embryo during Germination

**FIGURE 5:**
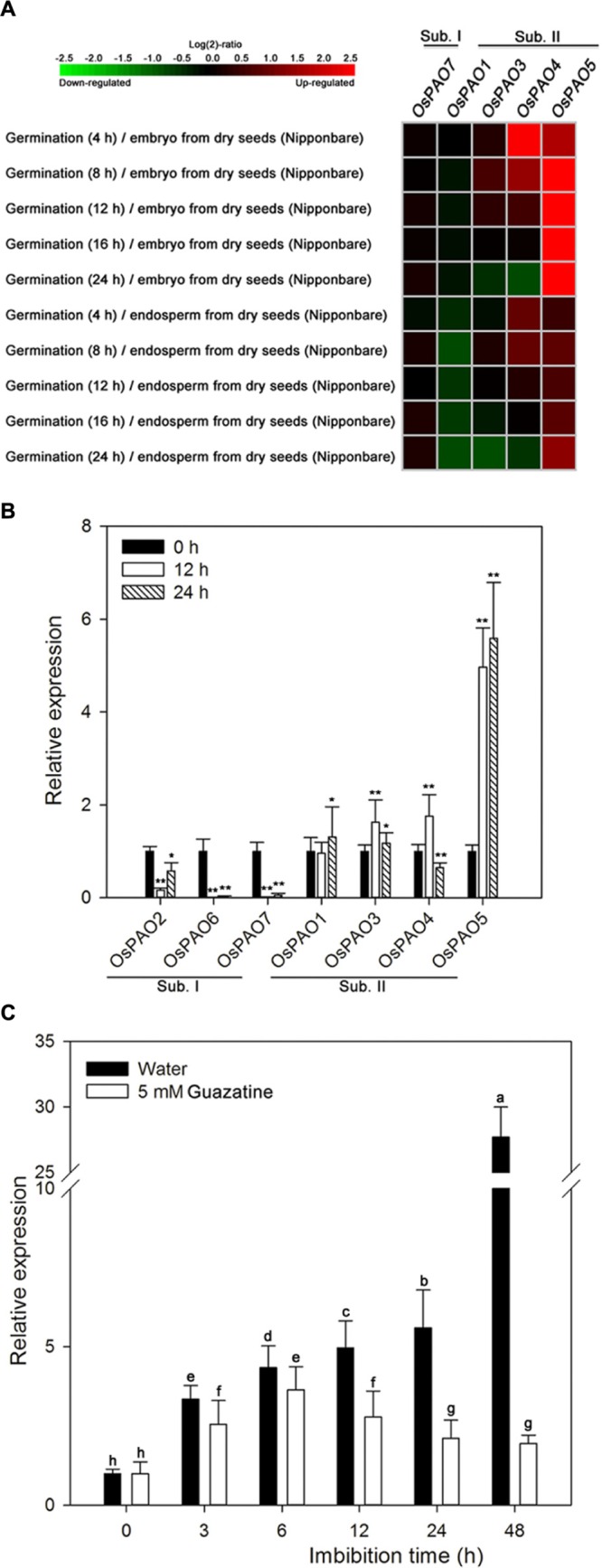
**Expression profiles of OsPAO genes during germination of rice seeds. (A)** Expression profiles (heat maps) obtained from rice microarray (Os_51k array) data as reported by GENEVESTIGATOR V3. Expression profiles for *OsPAO2* and *OsPAO6* were unavailable. The green/red coding reflects the relative expression levels with dark green representing strong downregulation and dark red representing strong upregulation. The corresponding numerical values are shown in Supplementary Table S2. **(B)** The expression levels of *OsPAO1∼7* in embryos assayed by qPCR after seeds had been imbibed in water for 0, 12, or 24 h. Data are the mean ± SE of three biological replicates of 30 embryos (∼0.1 g total). Significant differences for the qPCR data were assessed by the Student’s *t*-test (^∗^*P* < 0.05; ^∗∗^*P* < 0.01). **(C)** *OsPAO5* expression patterns in the embryo assayed by qPCR after seed imbibition of water or 5 mM guazatine at 0, 3, 6, 12, 24, and 48 h. Data are the mean ± SE of three biological replicates of 30 embryos (∼0.1 g total). Means denoted by the same letter did not significantly differ at *P* < 0.05 according to Fisher’s least significant difference test.

We examined the expression patterns of *OsPAO1/3–5/7*, by displaying the rice microarray data from the Genevestigator database as heat maps (**Figure 5A**) and found significant differences in the expression of these genes during germination as opposed to dry seeds. Moreover, the expression levels of these genes were distinctly different for the embryo and endosperm during germination. The *OsPAO5* expression levels were markedly upregulated (1.48–8.76 fold) in germinating seeds compared with those in dry seeds, whereas other *OsPAO* expression levels were not obviously different (**Figure 5A**; Supplementary Table S2).

To further assess the expression profiles of *OsPAO1–7*, qPCR was performed, and the results (**Figure 5B**) are consistent with the expression patterns from the microarray data. Transcription of *OsPAO5* was markedly upregulated in the embryos of germinating seeds compared with that in dry seeds, and the expression of *OsPAO6/7* was significantly downregulated, whereas the expression levels of the other genes were not obviously changed.

The expression profile of *OsPAO5* was then examined throughout the course of seed germination (at 0, 3, 6, 12, 24, and 48 h) by qPCR (**Figure 5C**). For seeds imbibed in water, the *OsPAO5* expression in the embryos progressively increased reaching its greatest value at 48 h. In contrast, for seeds imbibed in 5 mM guazatine, *OsPAO5* expression in the embryos increased until 6 h after which it decreased and seemed to be repressed by 12 h (**Figure 5C**). These results were consistent with those for PAO activity (**Figure 3**), changes in H_2_O_2_ content in embryos (**Figure 2**), and germination percentage in the rice seed (**Figure 1**), suggesting that *OsPAO5* is responsible for the PAO level and activity, and has an important role during germination of rice seeds.

## Discussion

### Involvement of H_2_O_2_ Generated by PAO Activity during Germination of Rice Seeds

For seeds of dicots, e.g., *Arabidopsis*, lettuce, and tomato, the endosperm cap is the main barrier to germination (Nonogaki et al., 2000; Müller et al., 2006; Zhang et al., 2014; Chen et al., 2016). Weakening of the cap and radicle elongation are required for completion of germination. However, for seeds of monocots, e.g., purple false brome, rice, barley, and maize, the main function of the endosperm is to provide nutritional energy for germination and seedling establishment (Bewley et al., 2013), and the coleorhiza is the main obstacle to radicle protrusion. Emergence of monocot radicles may depend on softening of the coleorhiza, and on the expansive force of the imbibing radicle cells (Gonzalez-Calle et al., 2015). For the study reported herein, we observed that the coleorhiza first protrude from the pericarp and then the radicle and coleoptile emerge from the coleorhiza during germination of rice seeds (**Figure 1**). Thus, the rice coleorhiza functions in a manner similar to that of the endosperm cap in dicot seeds. Endosperm cap weakening and radicle elongation during germination of dicot seeds require cell wall loosening, which involves both enzymes, e.g., mannase and cellulase, and non-enzymatic reactions, e.g., those of ROS (Nonogaki et al., 2010; Zhang et al., 2014; Scheler et al., 2015; Chen et al., 2016). These two types of reactions also appear to be required for germination of monocot seeds. For example, mannase activity has been detected in the coleorhiza and radicle during germination of rice and purple false brome seeds (Ren et al., 2008; Gonzalez-Calle et al., 2015). In our study, we found that the production and accumulation of H_2_O_2_ was greater in the coleorhiza and radicle than in the coleoptile of germinating rice seeds (**Figure 2**), indicating that H_2_O_2_ might be involved in the loosening of coleorhiza and radicle cell walls, which is a finding similar to what we found for germination in lettuce seeds (Zhang et al., 2014).

In addition to NADPH oxidases, PAOs and oxalate oxidases are enzymes that produce H_2_O_2_ (Cona et al., 2006; An et al., 2008). H_2_O_2_, O_2_^-^, and ^⋅^OH have been found to be involved in the loosening of cell walls (Müller et al., 2009). In the apoplast, ^⋅^OH, produced from O_2_^-^ and H_2_O_2_, may directly cleave wall polysaccharides to help destroy the integrity of the cell (Schweikert et al., 2000; Liszkay et al., 2004) and facilitate completion of germination (Zhang et al., 2014). We found that exogenous H_2_O_2_ promoted germination of rice seeds, whereas this process was inhibited by the H_2_O_2_ scavenger, DMTU (**Figure 1**; Ben Rejeb et al., 2015), suggesting that H_2_O_2_ is necessary for the germination process, a conclusion similar to that found for germination of sunflower seeds (Oracz et al., 2009). When the rice seeds were imbibed in water, the H_2_O_2_ content and PAO activity increased and paralleled that of the germination percentage. Conversely, for seeds imbibed in the PAO inhibitor, guazatine (**Figures 2** and **3**; Atanasov et al., 2016), germination was strongly inhibited, as was the H_2_O_2_ content and PAO activity in the embryos. These data indicate that PAO-produced H_2_O_2_ is essential for germination. Similar results by Zhang et al. (2011) demonstrated that PAO-produced H_2_O_2_ promotes germination of lettuce seeds. Moreover, much evidence supports the notion that PAO-generated H_2_O_2_ regulates such physiological processes as closure of fava bean stoma (An et al., 2008), development of soybean lateral roots (Su et al., 2006) and hypersensitive cell death of tobacco (Yoda et al., 2003, 2006). Additionally, the O_2_^-^ concentration and POD activity were increased in rice seeds when germinated in water (**Figure 2**), suggesting that H_2_O_2_, O_2_^-^, and ^⋅^OH are important to seed germination via their loosening of the coleorhiza cell walls of rice seeds (Schweikert et al., 2000; Liszkay et al., 2004; Müller et al., 2009). Interestingly, guazatine hardly inhibited the rate of O_2_^-^ production but intensely suppressed H_2_O_2_ production (**Figure 2**), which indicates that guazatine is not an effective inhibitor of O_2_^-^ production but is specific for H_2_O_2_ generation. Guazatine slightly suppressed POD activity (**Figure 2**), which might indirectly reflect the rate of ^⋅^OH production (Müller et al., 2009). Consequently, we speculate that a decrease in H_2_O_2_ production may reduce ^⋅^OH production and thereby inhibit germination of rice seeds.

### Functional Diversity of OsPAO Genes and the Possible Role(s) of OsPAO5 in the Germination of Rice Seeds

Gene duplication is often found to have occurred in eukaryotic genomes and thereby has contributed to biological diversity (Van de Peer et al., 2009; Magadum et al., 2013). Fusion of sequences encoding additional domains after gene duplication can lead to new functions associated with the duplicated gene products (Kaessmann, 2010). We identified 11 PAO homologs in the rice genome, which are distributed on chromosomes 1, 2, 4, 8, 9, and 10 (Supplementary Table S1). These proteins were classified into the four known and well-conserved subfamilies, I, IIa, IIb, and III, which have distinct subcellular locations, domain organizations, and functions (**Figure 4**). These observations suggest that a duplication of an ancestral PAO gene might have led to the expansion of the PAO gene family, which is associated with functional divergence. Unlike OsPAO1–7, members of subfamilies I, IIa, and IIb; *OsPAO8–11* encode histone lysine-specific demethylases, which are involved in control of flowering time and seed dormancy. The N-terminal SWIRM domain found in OsPAO8–11 may be the result of gene fusion, which may, therefore, have resulted in the functional diversity of rice PAO family members. This phenomenon is similar to what we found for NAD(H) kinase and NADPH oxidase (Scheler et al., 2015) family members (Li et al., 2014; Chang et al., 2016).

In plants, PAOs have diversified biochemical properties and physiological functions (Cona et al., 2006; Kusano et al., 2008; Moschou et al., 2008b; Alcázar et al., 2010; Angelini et al., 2010; Fincato et al., 2011; Wimalasekera et al., 2011). To begin with, PAOs were demonstrated the key enzyme for regulating cellular PAs levels which are critical for developmental processes, e.g., embryogenesis (Bertoldi et al., 2004; De-la-Pena et al., 2008), germination (Bethke et al., 2004; Liszkay et al., 2004), root growth (Cona et al., 2005), and flowering and senescence (Kakkar and Sawhney, 2002); for tolerance to stresses such as drought (Alcázar et al., 2006), salinity (Groppa and Benavides, 2008), temperature extremes (Groppa and Benavides, 2008), mineral deficiency, and wounding; and for defense against pathogens (Moschou et al., 2008c, 2009). Far from being only a means of regulating cellular polyamine levels, PAO contribute to important physiological processes through their reaction products [i.e., amino aldehydes, 1,3-diaminopropane (DAP) and hydrogen peroxide (H_2_O_2_)] that is we focus on. For example, PAs, DAP and H_2_O_2_ were the key signals associated with development, stress tolerance and defense in plants (Alcázar et al., 2010; Hussain et al., 2011; Wimalasekera et al., 2011). H_2_O_2_ derived from apoplastic catabolism of PAs acts synergistically with NO for the expression of defense and detoxification genes, and during hypersensitive reaction and developmental programmed cell death (Mittler et al., 2004; Moschou et al., 2008a; Wimalasekera et al., 2011).

Furthermore, PAO homologures includes PAOs, e.g., OsPAO1–7 and AtPAO1–5, and histone lysine-specific demethylases, e.g., OsPAO8–11 (OsHDMA701∼704) and AtPAO6∼9 (AtLDL1–3, AtFLD; **Figure 4**) with the first group catalyzing the terminal catabolism or back-conversion of PAs (Fincato et al., 2011; Kim et al., 2014; Liu et al., 2014a,b) and the second catalyzing the demethylation of histone H3K4 (Shi et al., 2004; Zhou and Ma, 2008; Zhao et al., 2015). These studies suggest that plant PAOs have multiple functions and are involved indirectly in developmental and physiological processes. The rice PAO homologs are divided into multiple subfamilies suggesting the functional diversity of these proteins as well.

Seed germination is a complex physiological and biochemical process that involves a series of signal transduction and regulation of gene expression (Bewley, 1997; Finch-Savage and Leubner-Metzger, 2006; Gomes and Garcia, 2013; He and Yang, 2013; Han and Yang, 2015). We confirmed that PAOs are involved in germination of rice seed and that they regulate H_2_O_2_ production via oxidative degradation of PAs (**Figures 1–3**). The PAO gene family in rice encodes 11 homologous proteins (**Figure 4**), but which of these protein(s) is important to germination was unclear prior to this report. Thus, we conducted microarray analysis and qPCR (**Figure 5**) to identify which of the seven PAOs (OsPAO1–7) is most important for germination. We found that *OsPAO5* is potentially the most important gene as its expression profile increased during the time seeds were imbibed in water. Transcription was somewhat inhibited by the specific PAO inhibitor guazatine as was accumulation of H_2_O_2_ and the PAO activity in the imbibed seeds.

Taken together, although major structural differences exist in seeds of monocot and dicot, the underlying mechanisms for regulation of seed germination seem similar as coleorhiza or endosperm weakening, respectively, and radicle elongation are required for all seed germination (Gonzalez-Calle et al., 2015). As with many dicot seeds, including those of lettuce (Zhang et al., 2014) and tomato (Morohashi, 2002), H_2_O_2_ specifically accumulates in the coleorhiza and radicle of the germinating rice seed. Moreover, PAOs, as enzymes that produce H_2_O_2_ by oxidation of PAs, are increasingly activity in imbibed rice seeds. The observed changes in germination percentage, H_2_O_2_ production, and PAO activity in the germinating rice seeds, suggest that H_2_O_2_ produced by PAOs is important to coleorhiza-limited germination. Given the functional classifications of the rice PAOs and the transcript expression of their genes during germination, *OsPAO5* probably is the gene that encodes most of the PAO activity that we observed during germination. Future studies should focus on the physiological role(s) of OsPAO5 and other OsPAOs, and the involvement of H_2_O_2_ and OsPAOs during germination, as they will help develop genetic methods, e.g., gene knockout and over-expression, that will increase our knowledge of how germination occurs.

## Author Contributions

B-XC and W-YL conceived and designed the experiments, analyzed the data and wrote the paper; B-XC and Y-TG performed all the experimental research and W-YL carried out bioinformatics analysis and provided funding; B-XC, W-YL, and Z-JC critically revised the manuscript; W-NZ offered the help for photography; Q-JL and ZC for the revision of the manuscript. All authors read and approved the final manuscript.

## Conflict of Interest Statement

The authors declare that the research was conducted in the absence of any commercial or financial relationships that could be construed as a potential conflict of interest.

## Footnotes

1 http://rapdb.dna.affrc.go.jp/index.html

2 http://www.arabidopsis.org/

3 http://croppal.org/

4 http://suba.plantenergy.uwa.edu.au/
