# Corrigendum: Involvement of Polyamine Oxidase-Produced Hydrogen Peroxide during Coleorhiza-Limited Germination of Rice Seeds

**DOI:** 10.3389/fpls.2017.02159

**Published:** 2017-12-12

**Authors:** Bing-Xian Chen, Wen-Yan Li, Yin-Tao Gao, Zhong-Jian Chen, Wei-Na Zhang, Qin-Jian Liu, Zhuang Chen

**Affiliations:** Argo-biological Gene Research Center, Guangdong Academy of Agricultural Sciences, Guangzhou, China

**Keywords:** seed germination, polyamine oxidases, hydrogen peroxide, *Oryza sativa*, *OsPAO5*, gene expression, *in silico* analysis

Jun Liu was one of the authors in the original article. At present, the author himself requested to remove his name from the authorship. The correct version of authorship appears above.

## Change in author contribution statement

B-XC and W-YL conceived and designed the experiments, analyzed the data and wrote the paper; B-XC and Y-TG performed all the experimental research and W-YL carried out bioinformatics analysis and provided funding; B-XC, W-YL, and Z-JC critically revised the manuscript; W-NZ offered the help for photography; Q-JL, ZC for the revision of the manuscript. All authors read and approved the final manuscript.

The authors apologize for these errors and state that this does not change the scientific conclusions of the article in any way.

The original article has been updated.

### Conflict of interest statement

The authors declare that the research was conducted in the absence of any commercial or financial relationships that could be construed as a potential conflict of interest.

