# Supplementary material for: Involvement of Polyamine Oxidase-Produced Hydrogen Peroxide during Coleorhiza-Limited Germination of Rice Seeds
Source: Front Plant Sci. 2016 Aug 12;7:1219. doi: 10.3389/fpls.2016.01219 (PMC4981591; doi:10.3389/fpls.2016.01219)
Supplement: Supplementary file 1 [file Table_1.PDF]

Table S1 The PAOs protein family genes in rice and *Arabidopsis**

Species*	Locus Name[#]	Gene Name[&]	GenBank Accession No.	A. A. length	Subcellular location[$]	E value[@]
Oryza sativa	Os01g0710200 (LOC_Os01g51320)	*OsPAO1* (*OsAO4*)	NM_001050573.1	512	Cytoplasm	4.30E-82
	Os03g0193400 (LOC_Os03g09810)	*OsPAO2* (*OsPAOa*)	NM_001055782.2	351	Extracellular	1.20E-36
	Os04g0623300 (LOC_Os04g53190/53195)	*OsPAO3* (*OsAO3*)	NM_001060458.1	484	Peroxisome	2.70E-90
	Os04g0671200 (LOC_Os04g57550)	*OsPAO4* (*OsAO1*)	NM_001060753.1	487	Peroxisome	3.50E-92
	Os04g0671300 (LOC_Os04g57560)	*OsPAO5* (*OsAO2*)	NM_001060754.1	492	Peroxisome	1.50E-88
	Os09g0368200 (LOC_Os09g20260)	*OsPAO6* (*OsPAOb*)	NM_001069545.2	496	Extracellular	2.40E-78
	Os09g0368500 (LOC_Os09g20284)	*OsPAO7*	NM_001069546.1	474	Extracellular	5.60E-76
	Os02g0755200 (LOC_Os02g51880)	*OsPAO8* (*OsHDMA702*)	NM_001054696.1	849	Nucleus	3.20E-87
	Os04g0560300 (LOC_Os04g47270)	*OsPAO9* (*OsHDMA701*)	NM_001060080.1	811	Plastid	9.50E-88
	Os08g0143400 (LOC_Os08g04780)	*OsPAO10* (*OsHDMA703*)	NM_001067511.1	763	Mitochondrion	5.00E-90
	Os10g0532100 (LOC_Os10g38850)	*OsPAO11* (*OsHDMA704*)	NM_001071681.1	1133	Nucleus	8.30E-94
Arabidopsis thaliana	AT5G13700	*AtPAO1*	NM_121373.3	472	Extracellular	4.30E-48
	AT2G43020	*AtPAO2*	NM_129863.3	490	Peroxisome	1.50E-93
	AT3G59050	*AtPAO3*	NM_115767.3	488	Peroxisome	1.30E-92
	AT1G65840	*AtPAO4*	NM_105256.3	497	Peroxisome	9.20E-95
	AT4G29720	*AtPAO5*	NM_119117.1	533	Cytoplasm	6.00E-107
	AT1G62830	*AtPAO6* (*AtLDL1*)	NM_104961.3	844	Nucleus	5.50E-94
	AT3G13682	AtPAO7 (*AtLDL2*)	NM_112218.1	746	Cytoplasm	2.60E-90
	AT4G16310	AtPAO8 (*AtLDL3*)	NM_117726.5	1628	Nucleus	7.30E-100
	AT3G10390	AtPAO9 (*AtFLD*)	NM_111874.4	884	Plastid	1.00E-89

* The data sources of each species are following: *Oryza sativa* (IRGSP v1.0, http://rapdb.dna.affrc.go.jp/) and *Arabidopsis thaliana* (TAIR v10.0, http://www.arabidopsis.org/).

[#] The corresponding MSU Ids of PAOs in rice were also showed in braces.

[&] The other names of PAOs in rice and *Arabidopsis* were also showed in braces.

[$] The subcellular locations of PAOs in rice and *Arabidopsis* were predicted by the crop Proteins with Annotated Locations (cropPAL, http://croppal.org/) and SubCellular Proteomic Database (SUBA3, http://suba.plantenergy.uwa.edu.au/), respectively.

[@] The E value of each PAO in hmmsearch by HMMER v3.0 with the Amine_Oxidase (PF01593) domain as HMM profiles.
