# Supplementary material for: Involvement of Polyamine Oxidase-Produced Hydrogen Peroxide during Coleorhiza-Limited Germination of Rice Seeds
Source: Front Plant Sci. 2016 Aug 12;7:1219. doi: 10.3389/fpls.2016.01219 (PMC4981591; doi:10.3389/fpls.2016.01219)
Supplement: Supplementary file 2 [file Table_2.PDF]

Table S2 The digital expression profiles of OsPAO family genes during rice seed germination*

PAO Genes / Germination seeds	*OsPAO7*		*OsPAO1*		*OsPAO3*		*OsPAO4*		*OsPAO5*	
	**Fold change**	**p-value**	**Fold change**	**p-value**	**Fold change**	**p-value**	**Fold change**	**p-value**	**Fold change**	**p-value**
**Germination (4 h) / embryo from dry seeds**	**1.08**	0.621	**-1.01**	0.898	**1.25**	0.191	**5.71**	0.002	**3.37**	0.002
**Germination (8 h) / embryo from dry seeds**	**1.04**	0.752	**-1.16**	0.095	**1.63**	0.013	**2.83**	0.004	**6.55**	<0.001
**Germination (12 h) / embryo from dry seeds**	**1.15**	0.417	**-1.16**	0.136	**1.36**	0.053	**1.58**	0.033	**7.49**	<0.001
**Germination (16 h) / embryo from dry seeds**	**1.06**	0.643	**-1.10**	0.133	**1.03**	0.750	**1.08**	0.759	**8.30**	<0.001
**Germination (24 h) / embryo from dry seeds**	**1.20**	0.282	**-1.13**	0.265	**-1.38**	0.091	**-1.66**	0.039	**8.76**	<0.001
**Germination (4 h) / endosperm from dry seeds**	**-1.09**	0.594	**-1.33**	0.043	**-1.10**	0.364	**2.00**	<0.001	**1.48**	0.007
**Germination (8 h) / endosperm from dry seeds**	**1.19**	0.321	**-1.67**	0.014	**1.22**	0.047	**1.97**	0.001	**1.93**	0.005
**Germination (12 h) / endosperm from dry seeds**	**1.03**	0.854	**-1.43**	0.021	**1.05**	0.454	**1.29**	0.212	**1.69**	0.008
**Germination (16 h) / endosperm from dry seeds**	**1.24**	0.34	**-1.51**	0.011	**-1.21**	0.142	**1.04**	0.609	**1.85**	0.006
**Germination (24 h) / endosperm from dry seeds**	**1.23**	0.077	**-1.68**	0.009	**-1.74**	0.005	**-1.40**	0.045	**2.70**	0.007

*, the corresponding expression profiles of PAO family genes in heat-maps were showed in Figure 5A.
