# Supplementary material for: Involvement of Polyamine Oxidase-Produced Hydrogen Peroxide during Coleorhiza-Limited Germination of Rice Seeds
Source: Front Plant Sci. 2016 Aug 12;7:1219. doi: 10.3389/fpls.2016.01219 (PMC4981591; doi:10.3389/fpls.2016.01219)
Supplement: Supplementary file 3 [file Table_3.PDF]

Table S3 The gene-specific primers used for qRT-PCR in this study

Gene Name	Primer Sequences	Amplicon Length	Span Introns / UTRs
OsPAO1	Forward: 5'-GTGTCCAAGATCCCGTGGTG-3'<br>Reverse: 5'-CCACGTAGCTGTATGACCCG-3'	256 bp	/
OsPAO2	Forward: 5'-GGTGAGCACACAAGCGAGTA-3'<br>Reverse: 5'-AGTCGTACTTGCCCTGAACG-3'	213 bp	Intron 7
OsPAO3	Forward: 5'-TGATTGCTCGAGGAAGGCAG-3'<br>Reverse: 5'-ACCAGACTCCCACACTCCAT-3'	128 bp	3'-UTR
OsPAO4	Forward: 5'-TGCCATTGTCATGGAATCTGGA-3'<br>Reverse: 5'-CATGCAGTTAATCCCCTACATGA-3'	110 bp	3'-UTR
OsPAO5	Forward: 5'-AGCCTGCCTATTTAGCACCG-3'<br>Reverse: 5'-TGTGAAACGGTACAGGATCTGA-3'	104 bp	3'-UTR
OsPAO6	Forward: 5'-CCATAGCACAGCTTCTTCCG-3'<br>Reverse: 5'-TGGCACCGATCTCCACATTG-3'	207 bp	Intron 1
OsPAO7	Forward: 5'-TCGCTACGAACATGACCAGC-3'<br>Reverse: 5'-TCTCGTCAATCACTAGAGCAGG-3'	133 bp	Intron 7, 3'-UTR
OsPAO8	Forward: 5'-ATGGAGGGTGATGAGCAACG-3'<br>Reverse: 5'-GCTCAGCGCTCACGACTATT-3'	188 bp	3'-UTR
OsPAO9	Forward: 5'-AGAAGCTTGACCGCATGATT-3'<br>Reverse: 5'-AAAGCCAGCGCTCAGTAGAA-3'	110 bp	3'-UTR
OsPAO10	Forward: 5'-CCAAGATCCTCCCTGCCATT-3'<br>Reverse: 5'-TCCAGAAGTGAGCTTATCCACC-3'	239 bp	3'-UTR
OsPAO11	Forward: 5'-ATGGCAAGCCACCGACATTA-3'<br>Reverse: 5'-TGAGCCCACCACGCATTTAT-3'	131 bp	/
*OsGAPDH1**	Forward: 5'-GCAATCAAGGAGGAGGCTGA-3'<br>Reverse: 5'-ACGTGTCGCTCAAAGCAATG-3'	139 bp	Introns 8, 9

*, *OsGAPDH1* (RAP-DB ID: Os02g0601300) was chosen as an internal control in rice.
