# Supplementary material for: Involvement of Polyamine Oxidase-Produced Hydrogen Peroxide during Coleorhiza-Limited Germination of Rice Seeds
Source: Front Plant Sci. 2016 Aug 12;7:1219. doi: 10.3389/fpls.2016.01219 (PMC4981591; doi:10.3389/fpls.2016.01219)
Supplement: Supplementary file 4 [file Image_1.PDF]

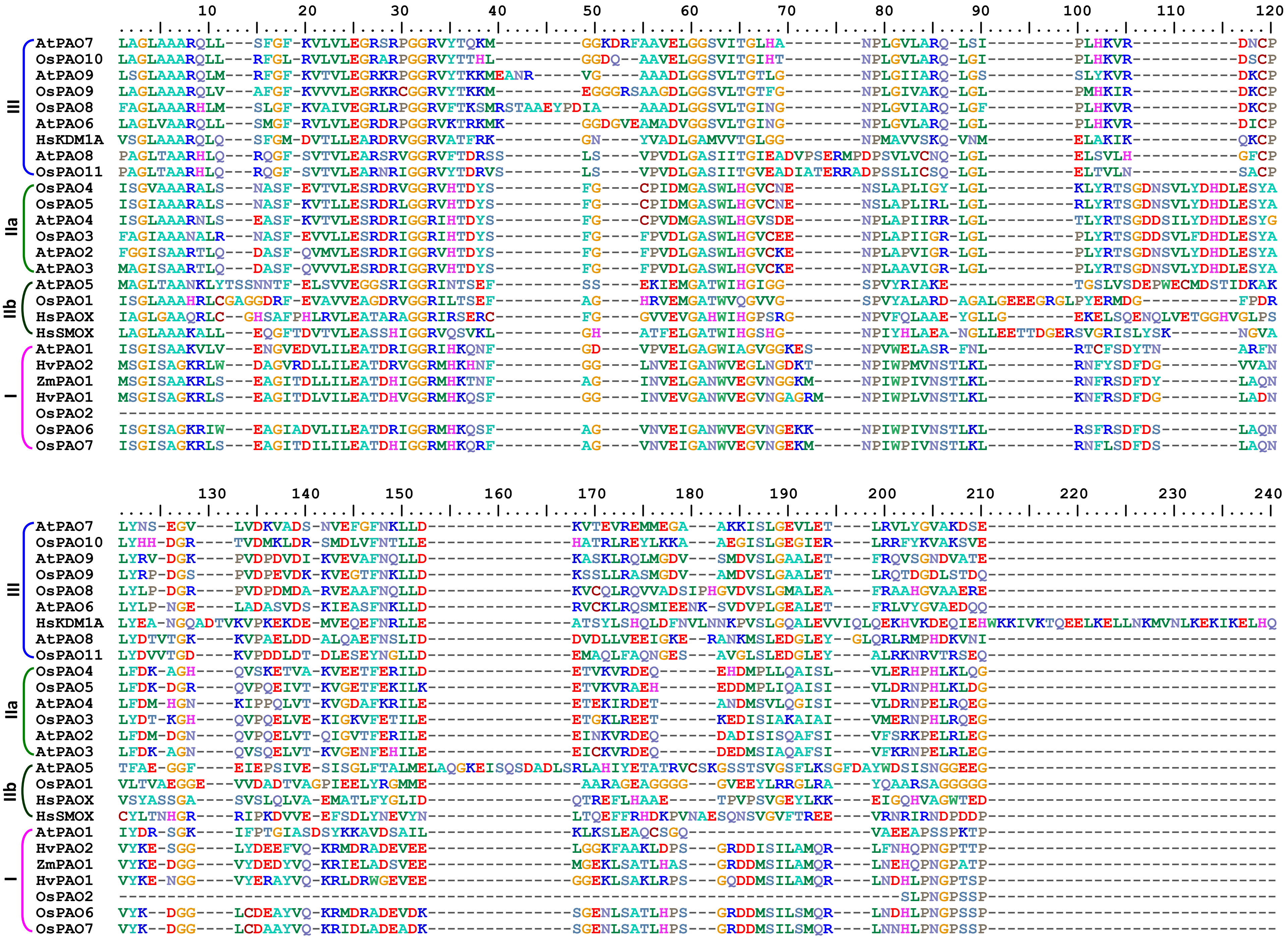
III
IIa
IIb
I
AtPAO7
OsPAO10
AtPAO9
OsPAO9
OsPAO8
AtPAO6
HsKDM1A
AtPAO8
OsPAO11
OsPAO4
OsPAO5
AtPAO4
OsPAO3
AtPAO2
AtPAO3
AtPAO5
OsPAO1
HsPAOX
HsSMOX
AtPAO1
HvPAO2
ZmPAO1
HvPAO1
OsPAO2
OsPAO6
OsPAO7

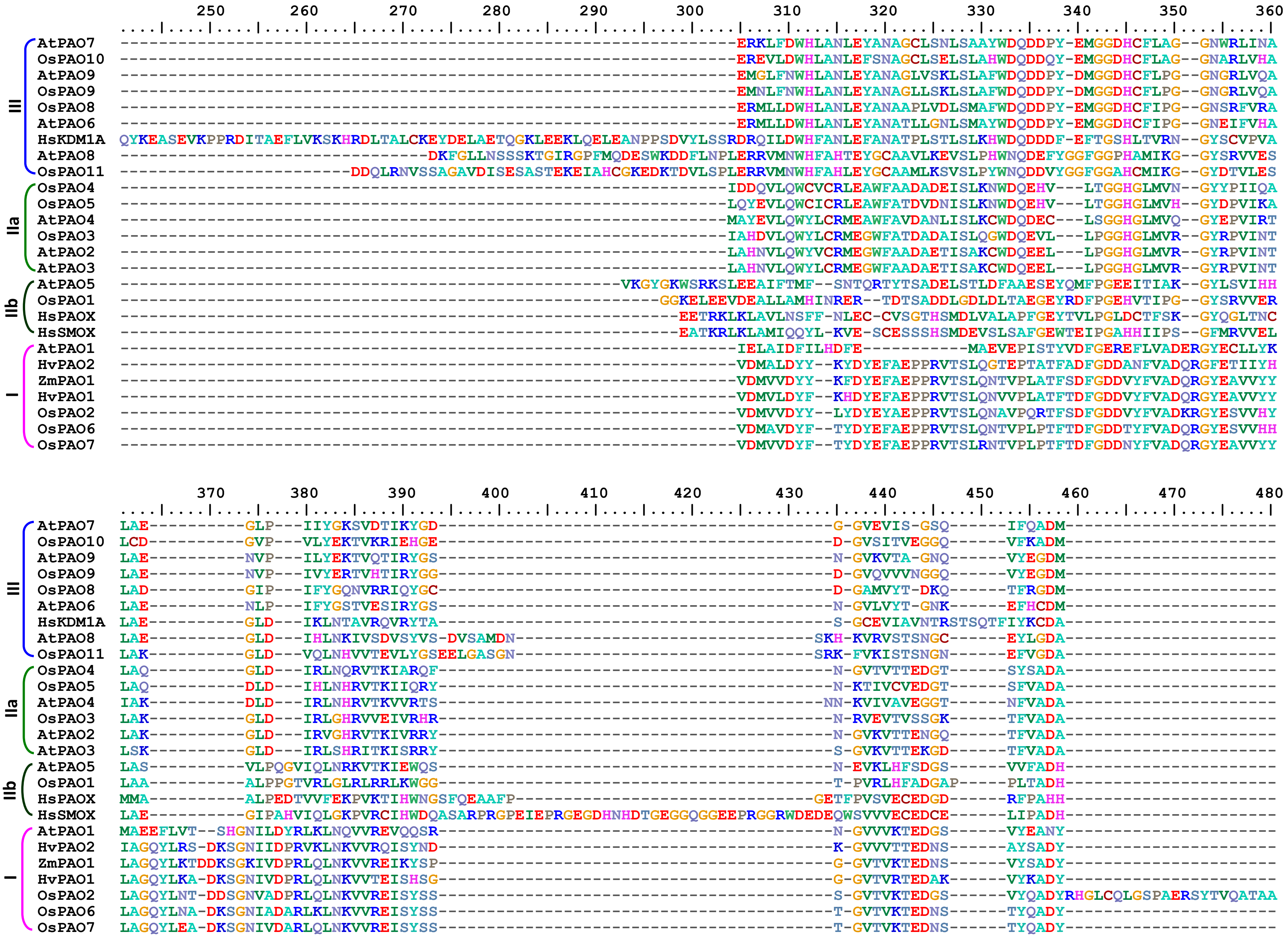
250 260 270 280 290 300 310 320 330 340 350 360
III
AtPAO7 --ERKLFDWHLANLEYANAGCLSNLSAAYWDQDDPY-EMGGDHCFLAG--GNWRLINA
OsPAO10 --EREVLDWHLANLEFSNAGCLSELSLAHWDQDDQY-EMGGDHCFLAG--GNARLVHA
AtPAO9 --EMGLFNWHLANLEYANAGLVSKLSLAFWDQDDPY-DMGGDHCFLPG--GNGRLVQA
OsPAO9 --EMNLFNWHLANLEYANAGLLSKLSLAFWDQDDPY-DMGGDHCFLPG--GNGRLVQA
OsPAO8 --ERMLLDWHLANLEYANAAPLVDLSMAFWDQDDPY-EMGGDHCFIPG--GNSRFVRA
AtPAO6 --ERMLLDWHLANLEYANATLLGNLSMAYWDQDDPY-EMGGDHCFIPG--GNEIFVHA
HsKDM1A QYKEASEVKPPRDITAEFLVKSKHRDLTALCKEYDELAETQGKLEEKLQELEANPPSDVYLSSRDRQILDWHFANLEFANATPLSTLSLKHWDQDDDF-EFTGSHLTVRN--GYSCVPVA
AtPAO8 --------------------------------DKFGLLNSSSKTGIRGPFMQDESWKDDFLNPLERRVMNWHFAHTEYGCAAVLKEVSLPHWNQDEFYGGFGGPHAMIKG--GYSRVVES
OsPAO11 ------------------------DDQLRNVSSAGAVDISESASTEKEIAHCGKEDKTDVLSPLERRVMNWHFAHLEYGCAAMLKSVSLPYWNQDDVYGGFGGAHCMIKG--GYDTVLES
IIa
OsPAO4 ---IDDQVLQWCVCRLEAWFAADADEISLKNWDQEHV---LTGGHGLMVN--GYYPIIQA
OsPAO5 ---LQYEVLQWCICRLEAWFATDVDNISLKNWDQEHV---LTGGHGLMVH--GYDPVIKA
AtPAO4 ---MAYEVLQWYLCRMEAWFAVDANLISLKCWDQDEC---LSGGHGLMVQ--GYEPVIRT
OsPAO3 ---IAHDVLQWYLCRMEGWFATDADAISLQGWDQEVL---LPGGHGLMVR--GYRPVINT
AtPAO2 ---LAHNVLQWYVCRMEGWFAADAETISAKCWDQEEL---LPGGHGLMVR--GYRPVINT
AtPAO3 ---LAHNVLQWYLCRMEGWFAADAETISAKCWDQEEL---LPGGHGLMVR--GYRPVINT
IIb
AtPAO5 --VKGYGKWSRKSLEEAIFTMF--SNTQRTYTSADELSTLDFAAESEYQMFPGEEITIAK--GYLSVIHH
OsPAO1 --GGKELEEVDEALLAMHINRER--TDTSADDLGDLDLTAEGEYRDFPGEHVTIPG--GYSRVVER
HsPAOX --EETRKLKLAVLNSFF-NLEC-CVSGTHSMDLVALAPFGEYTVLPGLDCTFSK--GYQGLTNC
HsSMOX --EATKRLKLAMIQQYL-KVE-SCESSSHSMDEVSLSAFGEWTEIPGAHHIIPS--GFMRVVEL
I
AtPAO1 --IELAIDFILHDFE-----------MAEVEPISTYVDFGEREFLVADERGYECLLYK
HvPAO2 --VDMALDYY--KYDYEFAEPPRVTSLQGTEPTATFADFGDDANFVADQRGFETIIYH
ZmPAO1 --VDMVVDYY--KFDYEFAEPPRVTSLQNTVPLATFSDFGDDVYFVADQRGYEAVVYY
HvPAO1 --VDMVLDYF--KHDYEFAEPPRVTSLQNVVPLATFTDFGDDVYFVADQRGYEAVVYY
OsPAO2 --VDMVVDYY--LYDYEYAEPPRVTSLQNAVPQRTFSDFGDDVYFVADKRGYESVVHY
OsPAO6 --VDMAVDYF--TYDYEFAEPPRVTSLQNTVPLPTFTDFGDDTYFVADQRGYESVVHH
OsPAO7 --VDMVVDYF--TYDYEFAEPPRVTSLRNTVPLPTFTDFGDDNYFVADQRGYEAVVYY
370 380 390 400 410 420 430 440 450 460 470 480
III
AtPAO7 LAE----------GLP---IIYGKSVDTIKYGD---G-GVEVIS-GSQ------IFQADM----------------------
OsPAO10 LCD----------GVP---VLYEKTVKRIEHGE---D-GVSITVEGGQ------VFKADM----------------------
AtPAO9 LAE----------NVP---ILYEKTVQTIRYGS---N-GVKVTA-GNQ------VYEGDM----------------------
OsPAO9 LAE----------NVP---IVYERTVHTIRYGG---D-GVQVVVNGGQ------VYEGDM----------------------
OsPAO8 LAD----------GIP---IFYGQNVRRIQYGC---D-GAMVYT-DKQ------TFRGDM----------------------
AtPAO6 LAE----------NLP---IFYGSTVESIRYGS---N-GVLVYT-GNK------EFHCDM----------------------
HsKDM1A LAE----------GLD---IKLNTAVRQVRYTA---S-GCEVIAVNTRSTSQTFIYKCDA----------------------
AtPAO8 LAE----------GLD---IHLNKIVSDVSYVS-DVSAMDN-------------------------------SKH-KVRVSTSNGC------EYLGDA----------------------
OsPAO11 LAK----------GLD---VQLNHVVTEVLYGSEELGASGN-------------------------------SRK-FVKISTSNGN------EFVGDA----------------------
IIa
OsPAO4 LAQ----------GLD---IRLNQRVTKIARQF---N-GVTVTTEDGT------SYSADA----------------------
OsPAO5 LAQ----------DLD---IHLNHRVTKIIQRY---N-KTIVCVEDGT------SFVADA----------------------
AtPAO4 IAK----------DLD---IRLNHRVTKVVRTS--NN-KVIVAVEGGT------NFVADA----------------------
OsPAO3 LAK----------GLD---IRLGHRVVEIVRHR---N-RVEVTVSSGK------TFVADA----------------------
AtPAO2 LAK----------GLD---IRVGHRVTKIVRRY---N-GVKVTTENGQ------TFVADA----------------------
AtPAO3 LSK----------GLD---IRLSHRITKISRRY---S-GVKVTTEKGD------TFVADA----------------------
IIb
AtPAO5 LAS----------VLPQGVIQLNRKVTKIEWQS---N-EVKLHFSDGS------VVFADH----------------------
OsPAO1 LAA----------ALPPGTVRLGLRLRRLKWGG---T-PVRLHFADGAP-----PLTADH----------------------
HsPAOX MMA----------ALPEDTVVFEKPVKTIHWNGSFQEAAFP-------------------------------GETFPVSVECEDGD------RFPAHH----------------------
HsSMOX LAE----------GIPAHVIQLGKPVRCIHWDQASARPRGPEIEPRGEGDHNHDTGEGGQGGEEPRGGRWDEDEQWSVVVECEDCE------LIPADH----------------------
I
AtPAO1 MAEEFLVT--SHGNILDYRLKLNQVVREVQQSR---N-GVVVKTEDGS------VYEANY----------------------
HvPAO2 IAGQYLRS-DKSGNIIDPRVKLNKVVRQISYND---K-GVVVTTEDNS------AYSADY----------------------
ZmPAO1 LAGQYLKTDDKSGKIVDPRLQLNKVVREIKYSP---G-GVTVKTEDNS------VYSADY----------------------
HvPAO1 LAGQYLKA-DKSGNIVDPRLQLNKVVTEISHSG---G-GVTVRTEDAK------VYKADY----------------------
OsPAO2 LAGQYLNT-DDSGNVADPRLQLNKVVREISYSS---S-GVTVKTEDGS------VYQADYRHGLCQLGSPAERSYTVQATAA
OsPAO6 LAGQYLNA-DKSGNIADARLKLNKVVREISYSS---T-GVTVKTEDNS------TYQADY----------------------
OsPAO7 LAGQYLEA-DKSGNIVDARLQLNKVVREISYSS---T-GVTVKTEDNS------TYQADY----------------------

```
                   490       500       510       520       530       540       550       560       570       580       590       600
III
AtPAO7    ----ILCTVPLGVLKK----------R--SIK-----FEPELPRRKQAAIDRLGFGLLNKVAMLFPSVFW--GDELDTF-----GCLNESSINRGEFFL----FY-------AYHTV-SG
OsPAO10   ----ALCTAPLGVLKS----------R--SII-----FEPELPERKLEAIQRLGFGLLNKVAMVFPHVFW--DEEIDTF-----GCLNKERSKRGEFFL----FY-------SYHTV-SG
AtPAO9    ----VLCTVPLGVLKN----------G--SIK-----FVPELPQRKLDCIKRLGFGLLNKVAMLFPYVFW--STDLDTF-----GHLTEDPNYRGEFFL----FY-------SYAPV-AG
OsPAO9    ----ALCTVPLGVLKN----------G--GVK-----FVPELPQRKLDSIKRLGFGLLNKVAMLFPHVFW--STDLDTF-----GHLTEDPSHRGEFFL----FY-------SYATV-AG
OsPAO8    ----VLCTVPLGVLKK----------G--NIQ-----FVPELPAQKREAIERLGFGLLNKVVLLFPYDFW--DGRIDTF-----GHLTEDSGQRGEFFL----FY-------SYSSV-SG
AtPAO6    ----ALCTVPLGVLKK----------G--SIE-----FYPELPHKKKEAIQRLGFGLLNKVAMLFPCNFW--GEEIDTF-----GRLTEDPSTRGEFFL----FY-------SYSSV-SG
HsKDM1A   ----VLCTLPLGVLKQ----------QPPAVQ-----FVPPLPEWKTSAVQRMGFGNLNKVVLCFDRVFW--DPSVNLF-----GHVGSTTASRGELFL----FW-------NLYKA---
AtPAO8    ----VLVTVPLGCLKA----------E--TIK-----FSPPLPDWKYASIKQLGFGVLNKVVLEFPTVFW--DDSVDYF-----GATAEETDLRGECFM----FW-------NVKKT-VG
OsPAO11   ----VLITVPLGCLKA----------Q--TIK-----FSPSLPDWKLSSIDRLGFGLLNKIVLEFPEVFW--DDNVDYF-----GATAEQTDLRGQCFM----FW-------NLKKT-VG
IIa
OsPAO4    ----CIITVPLGVLKA----------N--IIK-----FEPELPSWKSSAIADLGVGIENKIAMHFDTVFW---PNVEVL-----GMVGPTPKACG--------YFL------NLHKA-TG
OsPAO5    ----AIITVPLGVLKA----------N--IIK-----FEPELPDWKLSSISDLGIGIENKIALRFNSVFW---PNVEVL-----GRVAPTSNACG--------YFL------NLHKA-TG
AtPAO4    ----VIITVPIGVLKA----------N--LIQ-----FEPELPQWKTSAISGLGVGNENKIALRFDRAFW---PNVEFL-----GMVAPTSYACG--------YFL------NLHKA-TG
OsPAO3    ----AVIAVPLGVLKA----------N--TIK-----FEPRLPEWKEEAIRELSVGVENKIILHFSEVFW---PNVEFL-----GVVSSTTYGCS--------YFL------NLHKA-TG
AtPAO2    ----AVIAVPLGVLKS----------G--TIK-----FEPKLPEWKQEAINDLGVGIENKIILHFEKVFW---PKVEFL-----GVVAETSYGCS--------YFL------NLHKA-TG
AtPAO3    ----AVIALPLGVLKS----------G--MIT-----FEPKLPQWKQEAINDLGVGIENKIILNFDNVFW---PNVEFL-----GVVAETSYGCS--------YFL------NLHKA-TS
IIb
AtPAO5    ----VIVTVSLGVLKA-------------GIETDAELFSPPLPDFKSDAIRRLGYGVVNKLFVEMSQRKF---PSLQ-L-----VFDREDSEFRFVKIP----WWMRRTA--TITPIHSN
OsPAO1    ----VILTVSLGVLKASLGNKDTAGVGAAAIA-----FDPPLPPFKREAVARLGFGVVNKLFMEVEAVAPSEPEDVAGVQPAAAGFPFLHMAFRGHVSKIP--WWMRGTE--SICPVHAG
HsPAOX    ----VIVTVPLGFLRE----------HLDTF------FDPPLPAEKAEAIRKIGFGTNNKIFLEFEEPFW--EPDCQLI-----QLVWEDTSPLEDAAPELQDAWFRKLIGFVVLPAFAS
HsSMOX    ----VIVTVSLGVLKR----------QYTSF------FRPGLPTEKVAAIHRLGIGTTDKIFLEFEEPFW--GPECNSL-----QFVWEDEAESHTLTYPPELWYRKICGFDVLYPPERY
I
AtPAO1    ----VIVSASIGVLQS----------D--LLS-----FQPLLPRWKTEAIQKCDVMVYTKIFLKFPQCFWPCGPGQEFF-----IYAHEQRGYFT--------FWQ------HMENAYPG
HvPAO2    ----VMVSTSLGVLQS----------D--LIQ-----FKPQLPAWKIMAIYRFDMAVYTKIFLKFPKKFWPTGPGKQFF-----VYASSRRGYYG--------MWQ------SFEKEYPG
ZmPAO1    ----VMVSASLGVLQS----------D--LIQ-----FKPKLPTWKVRAIYQFDMAVYTKIFLKFPRKFWPEGKGREFF-----LYASSRRGYYG--------VWQ------EFEKQYPD
HvPAO1    ----VMVSTSVGVLQS----------D--LIQ-----FKPRLPTWKVLSIYQFDMAVYTKIFVKFPRKFWPQGKGREFF-----LYASSRRGYYG--------VWQ------EFEAQYPD
OsPAO2    CKFRVLCPARFSRIAS----------D--RCV-----LHVFDQKWKILAIYEFDMAVYTKIFVKFPKRFWPEGEGREFF-----LYASTRRGYYG--------IWQ------EFEKQYPD
OsPAO6    ----VMVSASLGVLQS----------D--LIQ-----FKPQLPSWKILAIYQFDMAVYTKIFVKFPKKFWPEGAGREFF-----LYASTRRGYYG--------VWQ------EFEKQYPD
OsPAO7    ----VMVSASLGVLQS----------D--LIQ-----FKPQLPSWKILAIYQFDMAVYTKIFVKFPKKFWPEGAGREFF-----LYASTRRGYYG--------VWQ------EFEKQYPD
```

```
                   610       620       630       640       650       660       670       680       690       700       710       720
III
AtPAO7    GPALVALVAGEAAQRFECTEPSVLLHRVLKKLRGIYGPK----------------GVVVPDPIQTVCTRWGSDPLSYGSYSHVRVGSSGVDYDILAESV-------SN------------
OsPAO10   GAVLIALVAGEAALEFEKVDPAVALHRVLGILKGIYGPK----------------GVTVPDPIQSCCTRWGSDPLCSGSYSHIRVGSSGTDYDILAESV-------ND------------
AtPAO9    GALLIALVAGEAAHKFETMPPTDAVTRVLHILRGIYEPQ----------------GINVPDPLQTVCTRWGGDPFSLGSYSNVAVGASGDDYDILAESV-------GDG-----------
OsPAO9    GPLLMALVAGEAAHNFETTPPTDAVSSVLKILRGIYEPQ----------------GIEVPDPLQSVCTRWGTDSFSLGSYSHVAVGASGDDYDILAESV-------GDG-----------
OsPAO8    GPLLIALVAGESAIEFEKTSPAENVEKVLETLRKIFSPK----------------GIEVPKPLQAICTRWGTDKFTYGSYSYVAIGSSGDDYDILAESV-------CD------------
AtPAO6    GPLLVALVAGDAAERFETLSPTDSVKRVLQILRGIYHPK----------------GIVVPDPVQALCSRWGQDKFSYGSYSYVAVGSSGDDYDILAESV-------GDG-----------
HsKDM1A   -PILLALVAGEAAGIMENISDDVIVGRCLAILKGIFG------------------SSAVPQPKETVVSRWRADPWARGSYSYVAAGSSGNDYDLMAQPI-------TPGPSIPGAPQPIP
AtPAO8    APVLIALVVGKAAFEYTNKSKSEHVNHAMMVLRKLFG------------------GDLVPDPVASVVTDWGTDPYSYGAYSYVAIGASGEDYDVLGRPV-------QN------------
OsPAO11   VPVLIALLVGKAAIDGQSISSDDHVKNAIVVLRKLFK------------------DASVPDPVASVVTNWGLDPFSRGAYSYVAVGASGRDYDILGRPV-------SD------------
IIa
OsPAO4    NPVLVYMAAGRFAQEVEKLSDKEAVDLVMSHLKKMLP--------------------DATEPTKYLVSRWGSDPNSLGSYSCDLVGKPADVSARFAAPV-------EN------------
OsPAO5    HPVLVCMVAGRFAYEFEKLSDEESVNFVMSQLKKMLP--------------------GATEPVQYLVSRWGTDPNSLGSYSCDLVGKPADLYERFCAPV-------GN------------
AtPAO4    HPVLVYMAAGNLAQDLEKLSDEATANFVMLQLKKMFP--------------------DAPDPAQYLVTRWGTDPNTLGCYAYDVVGMPEDLYPRLGEPV-------DN------------
OsPAO3    HPVLVYMPAGRLACDIEKLSDEAAAQFAFSQLKKILP--------------------NAAEPIHYLVSHWGSDENTLGSYTFDGVGKPRDLYEKLRIPV-------DN------------
AtPAO2    HPVLVYMPAGQLAKDIEKMSDEAAANFAVLQLQRILP--------------------DALPPVQYLVSRWGSDVNSMGSYSYDIVGKPHDLYERLRVPV-------DN------------
AtPAO3    HPVLVYMPAGQLARDIEKKSDEAAANFAFSQLQKILP--------------------DASSPINYLVSRWGSDINSLGSYSYDIVNKPHDLYERLRVPL-------DN------------
IIb
AtPAO5    SKVLLSWFAGKEALELEKLTDEEIKDAVMTTISCLTGKEVKNDTAKPLTNGSLNDDDEAMKITKVLKSKWGSDPLFRGSYSYVAVGSSGDDLDAMAEPLPKINKKVGQVNGHDQAKVHEL
OsPAO1    STVALAWFAGREAAHLESLPDDDVIRGAHATLDSFLP------------------AAPRWRVRRIKRSGWATDPLFLGSYSYVAVGSSGDDLDRMAEPLPR-----GPDAAADE-RPPSP
HsPAOX    VHVLCGFIAGLESEFMETLSDEEVLLCLTQVLRRVTG------------------NPRLPAPKSVLRSRWHSAPYTRGSYSYVAVGSTGGDLDLLAQPLP------ADGAGAQL------
HsSMOX    GHVLSGWICGEEALVMEKCDDEAVAEICTEMLRQFTG------------------NPNIPKPRRILRSAWGSNPYFRGSYSYTQVGSSGADVEKLAKPLP------YT----ESSKTAPM
I
AtPAO1    SNILVVTLTNEQSKRVEAQSDQETMKEAMSVLRDMFG-------------------ATIPYATDILVPRWWNNRFQRGSYSNYPMISDNQLLQNIKAPV-------GR------------
HvPAO2    ANVLLVTVTDVESRRIEQQPDNVTMAEAVGVLRNMFP------------------DRDVPDATDIYVLRWWSNRFFKGSYSNWPIGVNRYEYDQLRAPV-------GG------------
ZmPAO1    ANVLLVTVTDEESRRIEQQSDEQTKAEIMQVLRKMFP------------------GKDVPDATDILVPRWWSDRFYKGTFSNWPVGVNRYEYDQLRAPV-------GR------------
HvPAO1    ANVLLVTVTDDESRRIEQQSDNQTKAEIVEVLRSMFP------------------GEDVPDATDILVPRWWSDRFYRGTFSNWPIGVNRYEYDQLRAPV-------GR------------
OsPAO2    SNVLLVTVTDKESRRIEQQSDNQTKAEIMEVLRNMFP------------------DQDVPDATDILVPRWWSNRFYKGTFSNWPIGVNRYEYDQLRAPI-------ER------------
OsPAO6    ANVLLVTVTDEESRRIEQQPDSQTKAEIMEVVRCMFP------------------DEDVPDATDILVPRWWSDRFFRGSFSNWPIGVSRYEYDQLRAPV-------GR------------
OsPAO7    ANVLLVTVTDEESRRIEQQPDSQTKAEIMEVVRSMFP------------------DEDVPDATDILVPRWWSDRFFQGSFSNWPIGVSRYEHDQLRAPV-------GR------------
```

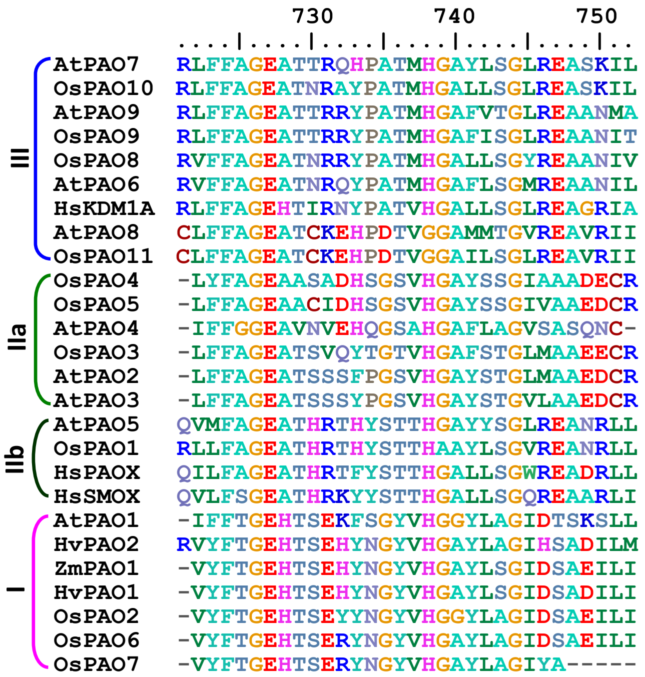

Figure S1. Amino acid sequences alignment analysis of Amino_oxidase domain of PAO proteins.

Amino acid sequences alignment analysis of Amino_oxidase (PF01593) in PAO genes from rice and *Arabidopsis*. The typical PAO protiens in human and other plants were also included, and their accession numbers used are as follows: HsPAOX (*Homo sapiens*, ENSP00000278060), HsSMOX (*H. sapiens*, ENSP00000307252), HsKDMA (*H. sapiens*, ENSP00000383042), ZmPAO1 (*Zea mays*, NM_001111636), HvPAO1/2 (*Hordeum vulgare*, AJ298131 and AJ298132). The four conserved PAO subfamilies are represented as I, IIa, IIb and III at the left side of the alignments.
